# What Can Domesticated Genes Tell Us about the Intron Gain in Mammals?

**DOI:** 10.1155/2012/278981

**Published:** 2012-05-30

**Authors:** Dušan Kordiš, Janez Kokošar

**Affiliations:** Department of Molecular and Biomedical Sciences, Josef Stefan Institute, Jamova 39, 1000 Ljubljana, Slovenia

## Abstract

Domesticated genes, originating from retroelements or from DNA-transposons, constitute an ideal system for testing the hypothesis on the absence of intron gain in mammals. Since single-copy domesticated genes originated from the intronless multicopy transposable elements, the ancestral intron state for domesticated genes is zero. A phylogenomic approach has been used to analyse all domesticated genes in mammals and chordates that originated from the coding parts of transposable elements. A significant amount of intron gain was found only in domesticated genes of placental mammals, where more than 70 cases were identified. *De novo* gained introns show clear positional bias, since they are distributed mainly in 5′ UTR and coding regions, while 3′ UTR introns are very rare. In the coding regions of some domesticated genes up to 8 *de novo* gained introns have been found. Surprisingly, the majority of intron gains have occurred in the ancestor of placental mammals. Domesticated genes could constitute an excellent system on which to analyse the mechanisms of intron gain. This paper summarizes the current understanding of intron gain in mammals.

## 1. Introduction

Transposable elements (TEs) constitute a major component of eukaryotic genomes [1]. Because they can transpose at high frequency they act as insertional mutagens and are powerful endogenous mutators [2, 3]. The mobility and amplification of TEs constitutes a major source of genomic variation either by virtue of their insertion or by triggering a variety of small- and large-scale chromosomal rearrangements. In consequence, they can have a major impact on the host phenotype [1–5]. Evidence is growing that TEs sometimes contribute positively to the function and evolution of genes and genomes [1–5]. Genome-scale analyses confirmed that domesticated or exapted TE-derived sequences have contributed diverse and abundant regulatory and protein coding sequences to host genomes [5–9]. 

Domesticated genes [6, 7, 9–11], originating from retroelements or from DNA transposons, constitute an ideal system for testing the hypothesis on the absence of intron gain in mammals. Since single-copy domesticated genes [7] originated from the intronless multicopy TEs [3], the ancestral intron state for domesticated genes is zero. Therefore, any intron present in these genes will constitute a *de novo* gained intron. The prerequisite for recognizing the origin, extent, and timing of *de novo* gained introns is reliable and wide taxon sampling [12]. In the past few years a quite large and dense collection of vertebrate, and especially mammalian, genomes has been accumulated. For some of these taxa a number of well-annotated genomes and genes exist, human and mouse genomes and transcriptomes being especially useful, with the full-length mRNAs that enabled reconstructions of the complete gene structures in these species [13, 14]. By using annotated human or mouse introns, we can trace their origin in mammals through genome-wide comparisons of orthologous genes in placentals, marsupials, and monotremes.

Spliceosomal introns are one of the major eukaryote-specific genome components, and the availability of numerous eukaryotic genomes has enabled genome-wide studies of the intron loss and gain dynamics [15–19]. The large-scale comparisons of the evolutionary dynamics of introns in eukaryotes has revealed a significant excess of losses and a nonuniform distribution of gains and losses [15–19]. A substantial excess of intron gains has been detected only for those intervals of eukaryotic evolution that are associated with major evolutionary innovations, such as the origin of eukaryotes and animals [15–19]. The large-scale comparisons of the evolutionary dynamics of introns have demonstrated surprising evolutionary stasis in the intron dynamics over the last 100–200 My [15, 16]. Large-scale intron studies in orthologous mammalian genes have indicated that very little intron turnover has occurred, with convincing evidence only for loss of introns [17, 18]. Such absence of intron gain in “recent” evolutionary history might be real, but could also be artifactual, the consequence of inadequate taxon sampling or inadequate comparisons, since only the “old” orthologous genes have been compared. To test the claims on the absence of intron gain in some taxonomic groups such as mammals [17, 18] and in recent evolutionary history (in the last 100–200 Mya) [15, 16], we need a quite simple and robust “gene model” that is independent of the inference procedures about intron gain. If the ancestral intron state is definitely known, the intron gain can be easily recognized. Such an approach, coupled with the known ancestral state (intronless), has been used in Kordis study [19] for evaluation of the hypotheses on the absence of intron gain in the recent evolutionary past [15, 16], and especially in mammals [17, 18].

## 2. Gene Structures of Domesticated Genes in Chordates and Mammals

Vertebrate, and especially mammalian, genomes contain a number of genes that have originated from TEs or their remains [6, 7, 9–11]. Since vertebrate retroelements and DNA transposons do not contain introns [3], the ancestral state for TE-derived genes is intronless. During the transition process from a multicopy TE to the single-copy domesticated gene, intron gain can occur, meaning that any intron present in the domesticated gene will constitute a *de novo* intron gain. An extensive phylogenomic analysis of all domesticated genes in chordate and mammalian genomes has therefore been made [19]. The rich collection of numerous mammalian genomes, belonging to all three major extant mammalian lineages, Eutheria (placentals), Metatheria (marsupials), and Prototheria (monotremes), was a major advantage in studying the origin and evolution of domesticated genes. By the phylogenomic analysis of all available domesticated genes in mammalian, vertebrate, and chordate genomes, unequivocal data about their origins (when and in which taxonomic group they originated) and numerous gene-related data (exon/intron structure, genome location, chromosomal position, etc.) have been obtained. The most important part of Kordis study has been the finding of transition point where and when TEs were transformed into domesticated genes, allowing *de novo* gain of introns to be precisely pinpointed in these genes. The gene structures of domesticated genes has provided direct evidence for extensive intron gain in placental mammals [19].

## 3. The Majority of Domesticated Genes Contain *De Novo* Gained Introns

The analysis of all known domesticated genes in chordates and mammals has shown that both retroelement- and DNA transposon-derived genes contain introns [19]. In the case of retroelement-derived genes the exon/intron structures are simple, since in these cases the process of gene fusion or exon shuffling to the preexisting “normal” genes is almost always absent (one such exception is the SCAND3 gene). However, the situation in the case of DNA transposon-derived genes is more complicated, since these genes can originate by three different routes: (a) from the entire DNA transposon, (b) by a complete DNA transposon being fused to the “normal” gene in the form of a single long exon that is 3′ end located, and (c) the most prevalent case, by gene fusion or exon shuffling of DNA binding domains (DBD) of DNA transposons with “normal” genes (where the exonization is necessary before the gene fusion). Therefore, in the case of DNA transposon-derived genes, intron gain can be recognized easily only in the first case, while the second and third cases are much more difficult for inferring intron gain in these genes. In the majority of the cases of fused entire transposases or just the DBDs, the newly recruited exons remain intact as very long or relatively short exons, but they are mostly without any intron. Therefore, in the case of DNA transposon-derived genes, these fused transposases and DBDs have been excluded from the analysis of intron gain. The situation regarding intron gain in retroelement-derived genes is definitely much simpler and less problematic, since no fusion genes have originated from retroelements (except SCAND3) [19].

## 4. The Burst of Intron Gain in Domesticated Genes Was in the Ancestor of Placental Mammals (Eutheria)

**Figure 1:**
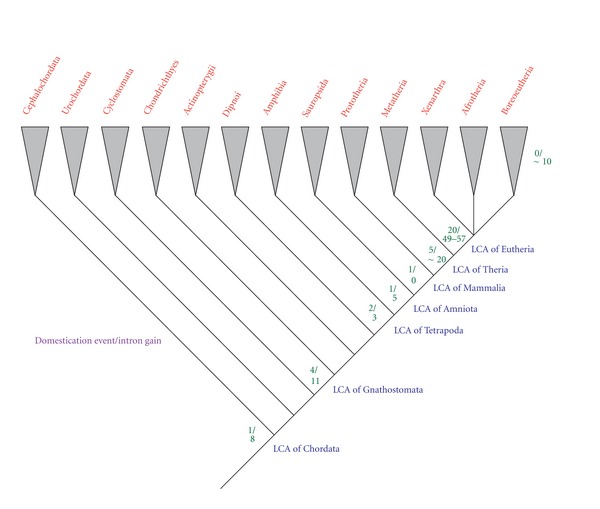
Numbers of transposable element-derived gene domestication events and intron gains mapped on the chordate phylogenetic tree. In the superorder Boreoeutheria some additional intron gains have occurred.

The analysis of all domesticated genes in chordates and mammals has shown that by far the greatest amount of intron gain occurred in the ancestor of placentals [19] (Figure 1). Twenty intron-containing domesticated genes originated in the ancestor of placentals, 18 of them being retroelement-derived and only two DNA transposon-derived genes. Interestingly, a recent study reported that 11 retrogenes with newly gained 5′ UTR introns also originated in the ancestor of placentals [20]. In the case of retrogenes they found 18 intron gains, 17 into the 5′ UTR, and a single gain in the 3′ UTR. In the case of domesticated genes 49 to 57 cases of *de novo* gained introns were found in the ancestor of placentals (Figure 1). In retroelement-derived genes 42 to 50 cases of intron gain have been found, while in DNA transposon-derived genes only 7 cases were found. Collectively, the 20 domesticated genes and 11 retrogenes provide evidence for at least 50 to 70 cases of intron gain in the ancestor of placentals [19]. This finding contrasts strongly with previous studies [17, 18], in which no intron gain could be found in mammals.

Although these genes represent a very small proportion of placental gene innovations, the observed extent of intron gain most probably represents just the tip of the iceberg. Regardless of the situation with the normal mammalian and vertebrate genes (“old genes”), there was a large-scale gene origination in the ancestor of placentals (evolutionarily young genes), at least in some classes of transcription factors (e.g., in C_2_H_2_ ZNFs). To test the extent of intron gain in some other placental-specific gene families the presence of intron gain has been analyzed in KRAB and SCAN ZNF genes [21, 22], especially in those orthologous genes that originated in the ancestor of placentals (>150 orthologous genes were analyzed). The analysis has shown that the amount of intron gain in these genes is not as high as in the case of TE-derived domesticated genes and retrogenes, but a number of cases with intron gain can, even so, be recognized [19]. 

The analysis of placental-specific domesticated genes, retrogenes and placental-specific transcription factors (~200 were analyzed) has shown that numerous intron gains occurred in the ancestor of placentals and that intron gain is still ongoing in mammals. At least 50 to 70 cases of intron gain have been documented from the analysis of >30 domesticated genes and retrogenes, and a few more cases have been documented also for placental-specific transcription factors (KRAB-ZNFs, SCAN-ZNFs and SCAN-KRAB-ZNFs). Up to 100 cases of intron gain have been recognized from the analysis of ~200 orthologous genes. These intron gains have occurred at different time points of placental evolution, the vast majority of them in the ancestor of placentals and the others in diverse lineages or species of placental mammals [19].

## 5. Numbers of Introns per Gene, Intron Densities, Sizes of Introns, and Preferred Locations of *De Novo* Gained Introns

An extensive phylogenomic analysis of all the domesticated genes in chordate and mammalian genomes has provided crucial information as to where and when TEs were transformed into domesticated genes, allowing *de novo* gain of introns in these genes to be pinpointed precisely [19]. The number of gained introns in these genes varies greatly, from 1 to 8. Domesticated genes in placentals and chordates accumulated a large number of introns, such that their density in these genes has become close to that in “normal” genes [23]. The average intron density for Eutheria-specific domesticated genes is 4.01 intron per kb of 5′ UTR. Intron density for older domesticated genes that originated early in vertebrates (e.g., Gin-1 and PGBD5) is 4.09 intron per kb of CDS. This comparison indicates that intron densities are similar in domesticated genes, the major difference however being in their position. Intron densities in Eutheria-specific domesticated genes and in older domesticated genes that originated early in vertebrates are therefore lower than those for normal mammalian and vertebrate genes [15, 16]. The sizes of the gained introns in domesticated genes are highly variable, ranging from a few hundred to a few thousand base pairs. DNA transposon-derived genes contain longer introns than retroelement-derived genes, just as evolutionarily older domesticated genes contain much longer introns than evolutionarily younger domesticated genes. Surprisingly, the longest introns exist in the gag-derived ZCCHC16 gene, and the second intron in the mouse (~410 kb long) is also the longest intron in the chordate domesticated genes [19]. This gene resembles mammalian retrogenes with very long introns [20]. The preferred locations of *de novo* gained introns in domesticated genes are the 5′ UTRs and coding regions, while 3′ UTR locations are very rare [19]. These preferred intron locations are similar to those of the “normal” chordate genes [24]. However, the preferred locations of *de novo* gained introns in domesticated genes differ from the mammalian retrogenes, where newly gained introns are preferentially located in the 5′ UTRs [20].

## 6. Intron Positions and Nucleotide Sequences of *De Novo* Gained Introns Are Highly Conserved in Placental Mammals

The extensive information on intron position conservation collected from the genomic alignments for all placental-specific intron gains has shown that the great majority of intron positions in placental-specific domesticated genes are highly conserved. The great majority of gained introns in domesticated genes have been fixed in the eutherian ancestor, as demonstrated by their presence and sequence conservation in all eutherian superorders [19]. From the genomic alignments we can readily trace the genes and their exons and introns compared at the nucleotide level over quite large evolutionary distances (at least 80–100 Myr) and, what is surprising, see remarkable level of conservation at the nucleotide level.

The rate of sequence divergence in introns is very high, therefore many introns are less conserved in sequence between organisms than their associated exons [25, 26]. The sequence conservation of *de novo* gained introns has been analyzed, and a striking conservation of the intron sequences in their entire length was found in 9 out of 19 retroelement-derived domesticated genes, showing 70–75% nucleotide identity between humans and Afrotheria, Xenarthra, and Laurasiatheria. Comparison of the entire domesticated genes between human and the representatives of all placental superorders has also shown ~75% nucleotide identity between humans and Afrotheria, Xenarthra, and Laurasiatheria [19]. Conservation of intronic sequences has been observed in some other Boreoeutheria genes [26], however only several short regions were shown to be highly conserved. The unusually conserved introns are mostly located in the 5′ UTR regions. It is possible that some of these introns are so highly conserved because they may have some conserved regulatory role in enhancing expression, in mRNA localization, stability, or efficiency of translation [27, 28]. It has been demonstrated that some of the domesticated genes are evolving under negative selection [10], therefore the level of unusual conservation is not limited to the exons but may also include the introns. Some of these genes are located on the X chromosomes, which may cause unusual patterns of evolution, such as lower mutation rates than on the autosomes [29]. The mutation rate on human X chromosome is indeed low and X-linked genes evolving mainly under negative selection are therefore evolving slowly [29, 30]. The analysis of intron conservation in other randomly selected genes indicates that intron sequences in all placental superorders may be more highly conserved than is generally acknowledged [19]. Such a high level of conservation of intron sequences may reflect their functional significance for the expression and regulation of domesticated and some other genes [27, 28].

## 7. Eutheria-Specific Domesticated Genes Are Alternatively Spliced

The analysis of domesticated genes in ASTD database (Alternative Splicing and Transcript Diversity Database; http://www.ebi.ac.uk/asd/index.html) has shown the presence of alternative splicing. It is interesting that DNA transposon-derived genes possess a larger amount of alternative splicing than the retroelement-derived genes. Up to 14 alternative splicing events can be seen per domesticated gene. More alternative splicing events can be seen in human than in mouse orthologous genes [19]. Alternative splicing in domesticated genes may have originated by mutations in splicing sites (evolution of weaker splice sites), by sequence changes in the intronic and exonic splicing silencers or enhancers (generating lower or higher densities) or by accumulation of *Alu* SINEs that can change the mode of splicing of the flanking exons [27, 31]. Since most of the alternative splicing events in domesticated genes are limited to humans the involvement of *Alu* SINEs is among the most interesting possibilities. The presence of alternative splicing events in humans indicates that these events might be quite recent. Although the gained introns in domesticated genes have been fixed in the eutherian ancestor, the alternative splicing events can be found, in the majority of cases, only in humans and, possibly, in primates. Such a distribution pattern may indicate very recent, and probably regulatory, adaptations in the human or primate lineages [19].

## 8. Lineage-Specific Enrichment of Intron Sequences with TEs in Diverse Placental Superorders

The majority of intron origination events in domesticated genes have occurred in the eutherian ancestor, but introns were later independently bombarded with lineage-specific TEs in all three eutherian sister groups Afrotheria, Xenarthra, and Boreoeutheria. Independent TE bombardment of introns occurred also inside Boreoeutheria, as evidenced by the large differences in TE repertoires in these introns between Laurasiatheria and Euarchontoglires, as well as between rodents and primates. Comparison of the orthologous introns in placental superorders has shown the presence of species- or lineage-specific enrichment of TEs and highly dynamic evolution of TE content in placental mammals [19]. These findings indicate that introns in each species are under constant bombardment with TEs [32]. By such accumulation of lineage-specific SINEs they may influence the alternative splicing of the flanking exons in some species [27, 31].

## 9. The Number of *De Novo* Gained Introns in Domesticated Genes is among the Highest in Eukaryotes

Genome-wide comparisons of closely related species in numerous intron-rich lineages have shown that recent intron gains are indeed very rare [15, 16, 33] and that intron losses outnumber intron gains in eukaryotic orthologous genes [15, 16]. Comparison of orthologous genes from mammalian genomes failed to reveal any intron gains at all, suggesting that all introns currently contained in mammalian genes were already present at the time of radiation of mammalian orders [17, 18]. However, in contrast to previous observations, Kordis study has demonstrated (based on the analysis of >200 orthologous genes) quite extensive intron gain, mainly in the ancestor of placental mammals. Therefore, the placental mammals can now be added to the list of taxonomic groups with significant amounts of intron gain arising in the relatively recent evolutionary past (100–200 Mya). Rates of intron gain in the past tens to hundreds of million years in diverse eukaryotes have been very low [15, 16, 25, 34]. Studies of closely related species have shown that diverse eukaryotic lineages experienced surprisingly few intron gains in this period (reviewed in [25, 34]). The highest rate of recent intron gain yet observed in genome-wide ortholog comparisons was in Oikopleura, where 4260 newly acquired introns have been detected [35]. As Kordis study has shown, the extent of intron gain in chordate, lower vertebrate, amniote, mammalian and therian ancestors has been much smaller. The domesticated genes have finally provided evidence for the numerous intron gains in the ancestor of placental mammals [19], more than 160 My ago [36]. At least 50–100 cases of intron gain have been observed in this ancestor. This extent of *de novo* gained introns is similar to that reported in diverse eukaryotic lineages [33, 34, 37, 38]. The comparative genomics of eutherian domesticated genes has shown differences in the numbers of introns, indicating that intron gain is still ongoing [19].

## 10. All Previous Claims for the Absence of Intron Gain in Mammals Were the Consequence of Inadequate Taxon Sampling and the Comparison of Only the “Old” Orthologous Genes

A substantial excess of intron gains has been detected only for those intervals of eukaryotic evolution that are associated with major evolutionary innovations, such as the origin of eukaryotes and animals [15, 16, 34]. The presence of ~100 intron gains in placental mammals is remarkable and clearly represents just the tip of the iceberg, the number of *de novo* gained introns in the ancestor of placental mammals probably being much higher. Kordis study pointed to the serious problems arising from comparison of orthologous introns in coding regions only and from sparse taxon sampling in the genome-wide analyses of intron gain [17, 18, 39]. None of the cases reported by Kordis were observed in the previous studies of closely related (human, mouse, rat and dog as an outgroup) [17] or distantly related (fish versus mammals) species [39]. In the closely related mammalian species analyzed [17, 18] intron gains occurred before those species originated. In comparisons of distantly related vertebrate species [39] only “old” orthologous genes have been compared, and evolutionary novelties were excluded from such analyses, however the neglected intron gains occurred after the analyzed species originated. Therefore, the overall extent of intron gain in eukaryotes could be much higher than reported in previous studies [19]. The solution to the above problems is to analyse the highly neglected evolutionary gene novelties at particular time points (like in the ancestor of placentals). Kordis study provides a further cautionary example in using only closely or distantly related species and sophisticated statistical methods in directionalizing intron loss/gain events, and underscores the importance of using appropriately selected taxa and evolutionary gene novelties for accurate inferences of genome evolution [19].

## 11. Intron Gain and Promoter Acquisition Are Intimately Linked in Domesticated Genes

The presence of numerous functional domesticated genes in mammals [6, 11] immediately raises the question of how they can obtain regulatory sequences that allow them to become transcribed—a precondition for gene functionality. To become expressed at a significant level and in the tissues where it can exert a selectively beneficial function, a new gene needs to acquire a core promoter and other structural elements that regulate its expression. Various sources of promoters and regulatory sequences exist and provide general insights into how new genes can acquire promoters and evolve new expression patterns [20, 40, 41]. The expression of domesticated genes may benefit from preexisting regulatory machinery and expression capacities of genes in their vicinity. Transcribed domesticated genes are often located close to other genes, suggesting that their transcription might be facilitated by open chromatin and/or regulatory elements of nearby genes. This possibility is supported by the observations that domesticated genes may be transcribed from the bidirectional CpG-rich promoters of genes in their proximity [42]. Some domesticated genes might also recruit CpG dinucleotide-enriched proto-promoter sequences in their genomic vicinity not previously associated with other genes for their transcription. Sometimes the promoters of domesticated gene may have evolved *de novo* through small substitutional changes under the influence of natural selection.

The process of promoter acquisition often involved the evolution of new 5′ untranslated exon-intron structures, which may span substantial distances between the recruited promoters and domesticated genes and is very similar to the situation observed in retrogenes [20]. Through the acquisition of new 5′-UTR structures, domesticated genes might also become transcribed from distant CpG-enriched sequences, which often have inherent capacity to promote transcription, and were not previously associated with other genes. These distant CpG “proto-promoter” elements might have been optimized by natural selection after they became associated with a functional domesticated gene. The frequent inheritance of CpG promoters might also help to explain why a significant number of domesticated genes evolved paternally or maternally imprinted expression [6, 11]. Thus, the primary role and selective benefit of newly gained 5′ UTR introns has been to span the substantial distances to potent CpG promoters driving transcription of domesticated genes and to reduce the size of the UTR exons.

## Abbreviations

DBD: DNA binding domain
LCA: Last common ancestor
Mya: Million years ago
My(r): Million year
TE: Transposable element
UTR: Untranslated region
ZNF: Zinc finger.
